# Contrasting population genetic structure of three semi‐terrestrial brachyuran crabs on the coast of the Japanese archipelago

**DOI:** 10.1002/ece3.11484

**Published:** 2024-06-05

**Authors:** Takeshi Yuhara, Hajime Ohtsuki, Shun K. Hirota, Yoshihisa Suyama, Jotaro Urabe

**Affiliations:** ^1^ Graduate School of Life Sciences Tohoku University Sendai Miyagi Japan; ^2^ Graduate School of Agricultural Sciences Tohoku University Osaki Miyagi Japan; ^3^ Botanical Gardens Osaka Metropolitan University Katano Osaka Japan; ^4^ Present address: National Institute for Environmental Studies Tsukuba Ibaraki Japan

**Keywords:** dispersal ability, genetic differentiation, genome‐wide SNP, ocean current, population genetics, semi‐terrestrial brachyuran crabs

## Abstract

Anthropogenic activities have reduced ecotones between the ocean and land, which is likely to threaten the population of brackish‐water brachyuran crabs. To assess the current status of these crabs, we examine the population genetic structures of three semi‐terrestrial brachyuran crabs widely distributed along the coast of the Japan and to clarify factors determining their genetic structures. We collected 184 *Orisarma dehaani*, 252 *Chiromantes haematocheir*, and 151 *Helice tridens* crabs from 36 localities of the Japanese archipelago. Genome‐wide SNP data from these crabs were analyzed using MIG‐seq. Bayesian clustering of STRUCTURE and DAPC analysis were used to identify genetically disturbed populations and to visualize genetic differentiation between local populations. Genetic population structure showed clear differentiation between populations on the Pacific coast of the Tohoku region and on other Japanese coasts in *O*. *dehaani*, but not in *C*. *haematocheir* or *H*. *tridens*. The inbreeding coefficient of *O*. *dehaani* was significantly higher on the Pacific coast of the Tohoku region compared to other Japanese coasts. *C*. *haematocheir* and *H*. *tridens* had homogeneous genetic structures along the Japanese coast, but showed genetic differentiation of a local population at their range limits. Thus, *O. dehaani* showed little gene flow and clear genetic differentiation between populations in the Tohoku Pacific region and those on other Japanese coasts due to ocean currents. Although such a regional differentiation was not found in *C*. *haematocheir* and *H*. *tridens*, one population of *C*. *haematocheir* was genetically isolated at the edge of its distribution range and likely vulnerable to environmental changes.

## Graphical abstract

This study highlights the potential extinction threat faced by semi‐terrestrial crabs. We analyzed the population genetics of these species distributed along the coast of the Japanese archipelago using modern high‐resolution genome‐wide SNP analysis. We found that one crab species had clear spatial genetic differentiation among populations, despite its ability to disperse long distances during larval stages. In addition, a population of another species was isolated at the edge of its range. Based on these results, we emphasized the need to conserve local populations of these species, as their local populations have evolved independently.

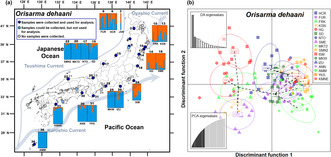

## 1 INTRODUCTION

In recent decades, the development of coastal regions by human activities has altered and reduced the ecotone areas between the ocean and land. These areas are the habitats of brackish‐water and semi‐terrestrial brachyuran crabs (Miyake, 1983). Accordingly, the populations of these crabs may be threatened with extinction. Therefore, it is necessary to assess the current status of the brackish‐water brachyuran crab populations.

Studies have shown that endangered populations tend to have low genetic diversity and become genetically isolated from populations in other areas (Frankham et al., 2010). Indeed, in addition to anthropogenic activities, various factors, such as the dispersal ability of the planktonic larval stages (DeWoody & Avise, 2000; Palumbi, 1992) and ocean currents as invisible physical barriers (Gilg & Hilbish, 2003; Palumbi, 1992), are known to affect the population genetic structure of coastal invertebrates. In the Japanese archipelago, several ocean currents, including Oyashio, Kuroshio and Tsushima currents, are known to genetically differentiate the local populations of coastal organisms, such as gobies (Matsui, 2022), snails (Kojima et al., 2004; Nakano et al., 2010; Yamakawa & Imai, 2014) and the seaweeds (Hu et al., 2016; Zhong et al., 2020). These organisms have phylogeographic patterns composed of “Pacific Ocean lineage” and “Sea of Japan lineage,” which correspond to the paths of the two currents, the Kuroshio Current and the Tsushima Current, respectively (Matsui, 2022). Although several studies have examined the genetic population structure of brachyuran crabs, such as the varunid crab *Ptychognathus ishii* (Kawane & Wada, 2015), the sesarmid crab *Clistocoeloma sinense* (Yuhara et al., 2014), the camptandriid crabs *Deiratonotus cristatus* (Kawamoto et al., 2012) and *Deiratonotus kaorie* (Kawane et al., 2012), and the ocypodid crabs *Austruca lactea* (Tokuyama et al., 2020) and *Tubuca arcuata* (Aoki et al., 2008), the factors affecting their genetic structure are not well understood, since the distribution ranges of these crabs are geographically restricted to the limited area of the Japanese coast.

The sesarmid crabs *Orisarma dehaani* and *Chiromantes haematocheir*, and the varunid crab *Helice tridens* inhabit the coastal land‐ocean ecotone and are endemic to East Asia (Japan, Korea, China, and Taiwan) (Miyake, 1983; Sakai, 1976; Sakai et al., 2006; Shih, 2007). These semi‐terrestrial crabs have planktonic zoeal larval stages (Cuesta et al., 2006) that can spatially disperse using ocean currents to expand their range in coastal areas. Adult individuals of these three crab species live primarily in estuaries and salt marshes. However, their habitats differ slightly each other (Miyake, 1983; Sakai, 1976): *C*. *haematocheir* inhabits mainly inland forest areas, *O*. *dehaani* occurs mainly in areas close to water, such as wetlands, and *H*. *tridens* inhabits muddy shores of the mid to high intertidal zone. In addition, these species are known to have different settlement patterns, presumably due to different preference for salinity and temperature (Matsumoto et al., 2020; Saigusa, 1981).

In East Asia, the Japanese archipelago is surrounded by coasts on all sides and has a very long coastline of about 30,000 km (Fujikura et al., 2010). As these semi‐terrestrial crabs are widely distributed along the coastline of the Japanese archipelago (Miyake, 1983), they are ideal organisms to study the factors affecting the population genetic structure of brachyuran crabs because these crabs, which inhabit in the land‐ocean ecotone, are often classified as locally endangered species in Japan (Japanese Association of Benthology, 2012). In this study, therefore, we examined the population genetic structures of these three crabs in the Japanese archipelago to clarify whether they are genetically differentiated among the geographically distinct areas and, if so, what factors caused these genetic differentiations. Specifically, we analyzed samples collected from 36 sites in the Japanese archipelago using sequence data of mtDNA COI sequence and multiplexed inter‐simple sequence repeat (ISSR) SNP data. We then used these data to assess whether there were local populations threatened with extinction.

## 2 MATERIALS AND METHODS

### 2.1 Specimens

The three semi‐terrestrial crabs were collected as whole bodies by hand‐digging in 2019 and 2020 from a total of 36 tidal flats and salt marshes across the four major islands of the Japanese archipelago (Figure S1, Table S1). We collected a total of 184 individuals of the sesarmid crab *Orisarma dehaani* from 21 of 43 localities and 252 individuals of the sesarmid crab *Chiromantes haematocheir* from 26 localities. We also collected a total of 151 individuals of the varunid crab *Helice tridens* from 16 localities. These specimens were either frozen at −20°C or stored in 95% ethanol until DNA extraction.

### 2.2 DNA extraction

Genomic DNA was extracted using a DNeasy Blood & Tissue Kit (QIAGEN). The concentration of gDNA was measured using a “NanoDrop™ Lite” spectrophotometer (Thermo Scientific, Waltham, MA) and was adjusted to 1 ng/mL for PCR amplification.

### 2.3 Mitochondrial DNA

In each specimen, the target DNA segment of a portion of the mtDNA cytochrome c oxidase subunit I (COI) was amplified using polymerase chain reaction (PCR) with the primers mtd10 5′‐TTGATTTTTTGGTCATCCAGAAGT‐3′ (Roehrdanz, 1993) and C/N2769 5’‐TTAAGTCCTAGAAAATGTTGGGGA‐3′ (Gopurenko et al., 1999). PCR amplification was performed in a total volume of 20 mL containing 0.16 mL of TaKaRa ExTaq™ (5 units/mL), 2.0 mL of 10× Ex Taq buffer, 1.6 mL of dNTP mixture (2.5 mM each), 1.0 mL of each primer (10 mM), and 2.0 mL of template. The PCR conditions included 35 cycles of denaturation (94°C, 30 s), annealing (45°C, 30 s), and extension (72°C, 60 s) on a T100 Thermal Cycler (Bio‐Rad). All amplified samples were purified using ExoSAP‐IT® (Affymetrix) and subsequent product sequencing was performed at Eurofins Genomics. The obtained DNA sequences were deposited in DDBJ (accession numbers LC768188 – LC768321).

### 2.4 MIG‐seq

Genome‐wide SNP data were analyzed using the MIG‐seq method (Suyama & Matsuki, 2015) using DNA samples from 1 to 10 individuals collected at each location in each species, i.e., 154 individuals collected at 18 locations for *O. dehaani*, 183 individuals collected at 22 locations for *C. haematocheir*, and 145 individuals collected at 16 locations for *H. tridens*. A MIG‐seq library was constructed by two step PCR as described by Suyama et al. (2022). Inter‐simple sequence repeat (ISSR) regions were amplified by first PCR using MIG‐seq primer set‐1 (Suyama & Matsuki, 2015). The PCR products were checked using a Microchip Electrophoresis System (MultiNA, Shimadzu, Japan) with a DNA2500 Reagent Kit (Shimadzu), which was also used to quantify the second‐round PCR products. In the subsequent steps for a second PCR, the complementary sequences for the binding sites of Illumina sequencing flow cell and the indices (barcodes) for each sample were added to the first round PCR products. The second PCR products were mixed and extracted ≥350 bp fragments. Sequencing was performed by Illumina MiSeq sequencers (MiSeq Control Software ver. 2.0.12; Illumina, San Diego, CA) using a MiSeq Reagent Kit ver. 3 (150 cycle; Illumina) with 80‐bp paired‐end reads. The MIG‐seq data was deposited at the DDBJ Sequence Read Archive (DRA, accession number DRA016447).

Extremely short reads containing adapter sequences and low‐quality reads were removed using Trimmomatic 0.39 (Bolger et al., 2014) with the following parameters, ‘ILLUMINACLIP:adapter.fasta:2:30:10 CROP:77 HEADCROP:6 SLIDINGWINDOW:4:15 MINLEN:71’ (a FASTA format file, adapter.fasta, contains Illumina adapter sequences: CAGAGATCGGAAGAGCGTCGTGTAGGGAAAGA, GTCAGATCGGAAGAGCACACGTCTGAACTCCAGTCAC). Stacks 2.62 pipeline was used for de novo SNP genotyping (Rochette et al., 2019) with the following parameters: minimum depth of coverage required to create a stack (*m*) = 3, maximum distance between stacks (*M*) = 2, maximum mismatches between loci when building the catalog (*n*) = 2. SNP selection was performed using populations program of Stacks. We selected only SNPs with more than 60% coverage among samples from each crab for MIG‐seq. In addition, SNPs where one of two alleles had less than 5% was filtered out. SNPs with high heterozygosity (*H*o ≥ 0.6) were also removed. To avoid linked SNP, we used only first SNP per locus.

### 2.5 Data analysis

The number of mtDNA haplotypes, and the haplotype‐ and nucleotide‐diversity were calculated for each population of the three semi‐terrestrial crabs using the computer software programs Arlequin ver. 2.001 (Schneider et al., 2000) and DnaSP 5.10.1 (Librado & Rozas, 2009). Phylogenetic relationships among the haplotypes were inferred for each species using the statistical parsimony software TCS Networks (Clement et al., 2002) implemented in the package PopART (http://popart.otago.ac.nz). The alignment data were made with the default settings in MUSCLE implemented in MEGAX (Kumar et al., 2018).

To identify genetically distinct populations, we performed Bayesian clustering using STRUCTURE ver. 2.3.4 software (Pritchard et al., 2000), which assigns individuals into K clusters. The population structure was simulated with values of *K* = 1–10 under an admixture model, i.e., the correlated allele frequency model (Hubisz et al., 2009). All runs included 100,000 Markov chain Monte Carlo generations after a burn‐in period of 100,000 iterations. Ten runs were performed for each value of *K*. The optimal *K* value was determined using the ΔK methods of Evanno et al. (2005) in STRUCTURE Harvester program (Earl & Vonholdt, 2012, http://taylor0.biology.ucla.edu/structureHarvester/). Detection of genetically distinct populations was done by the analysis of SNP data using STRUCTURE software (Pritchard et al., 2000). For species that showed clear genetic differentiation between populations (optimal *K* > 1) in the STRUCTURE, we performed a discriminant analysis of principal components (DAPC; Jombart et al., 2010) to visualize between‐population differentiation in the SNPs. This analysis extracts information from the data by first performing a principal component analysis on user‐defined populations. Then, using the PCA factors as variables, a discriminant analysis is performed to maximize the inter‐population component of variation. In the present study, the DAPC was implemented using the optimized number of principal components as determined by the a‐score and conducted using the “adegenet” package (Jombart et al., 2010) for the statistical platform R 4.03 (R Core Team, 2020) after the text data file was converted to the Genepop file format. The number of polymorphic SNPs observed, and the expected heterozygosity for each population were calculated using POPULATIONS in Stacks (Catchen et al., 2013; Rochette et al., 2019). Fixation index (*F*_IS_) for each population was calculated and their significance was tested by 1000 permutations using FSTAT 2.9.4 (Goudet, 2003). Phylogenetic relationships among the populations of *O*. *dehaani* were assessed by constructing a neighbor‐joining network based on DA distances between the populations (Nei et al., 1983) using SplitTree 4 (Bryant & Moulton, 2004). Statistically significant difference nodes were evaluated from bootstrap probabilities based on 1000 replicates of the neighbor‐joining method using POPTREE2 (Takezaki et al., 2010).

### 2.6 Estimation of spatial genetic structure and migration patterns

Using the SNP data obtained for each of the three semi‐terrestrial crabs, pairwise population *F*_ST_ values were estimated by the AMOVA using the GenAlEx 6.5 (Peakall & Smouse, 2012). Since missing data can be particularly problematic for pairwise distance‐based analyses, large numbers of missing data should be minimized (Blyton & Flanagan, 2012). For this analysis, we used a dataset consisting of 36 loci in *O*. *dehaani*, 19 loci in *C*. *haematocheir*, and 47 loci in *H*. *tridens* with 90%, 90% and 95% sharing sites, respectively. Statistical significance levels for all pairwise tests were set at 0.05 after adjusting for multiple comparisons using the Bonferroni correction (Rice, 1989) in this analysis. To evaluate genetic differentiation at the population level, we also measured the geographic coastal distance between each sampling locality along the coastline using distances between Japanese seaports (The Japan Shipping Exchange, Inc., 1996). For each of three semi‐terrestrial crabs, a Mantel test (Mantel, 1967) was performed to assess correlations between genetic differentiation (*F*_ST_ /(1− *F*_ST_)) and geographic distance with 9999 permutations in the “ade4” package (Dray & Dufour, 2007) for R 4.03 (R Core Team, 2020).

Finally, the gene flow between the populations of *O*. *dehaani* was estimated by using divMigrate‐Online (https://popgen.shinyapps.io/divMigrate‐online/). This program generates a migration network graph with relative values for gene flow among populations scaled to the largest magnitude estimated. For the analysis, we selected two populations obtained from the K clusters of the STRUCTURE analysis. We used *N*m as a measure of genetic distance. The significance of asymmetric gene flow between populations was tested by 1000 bootstrap iterations.

## 3 RESULTS

### 3.1 Orisarma dehaani

#### 3.1.1 Mitochondrial DNA

DNA segments of 498 bp in the mitochondrial COI gene were sequenced and used for analyses. No insertions or deletions were found in these segments. In *Orisarma dehaani*, 48 different mtDNA haplotypes were identified from a total of 50 variable sites in the segments. Sixteen of the 48 haplotypes were shared among 2 to 19 localities, while the remaining 29 haplotypes were specific to single localities (Table S2). Haplotype diversity values (*h*) and nucleotide diversity values (*π*) ranged from 0.607 to 1.000 and from 0.0022 to 0.0079, respectively. According to a statistically parsimonious haplotype network (Figure S2a), the dominant haplotype was Od1, and the other haplotypes were separated from Od1 by 1 to 7 mutational steps, and formed a “bush‐like” shape (Figure S2a,b).

#### 3.1.2 MIG‐seq

**FIGURE 1:**
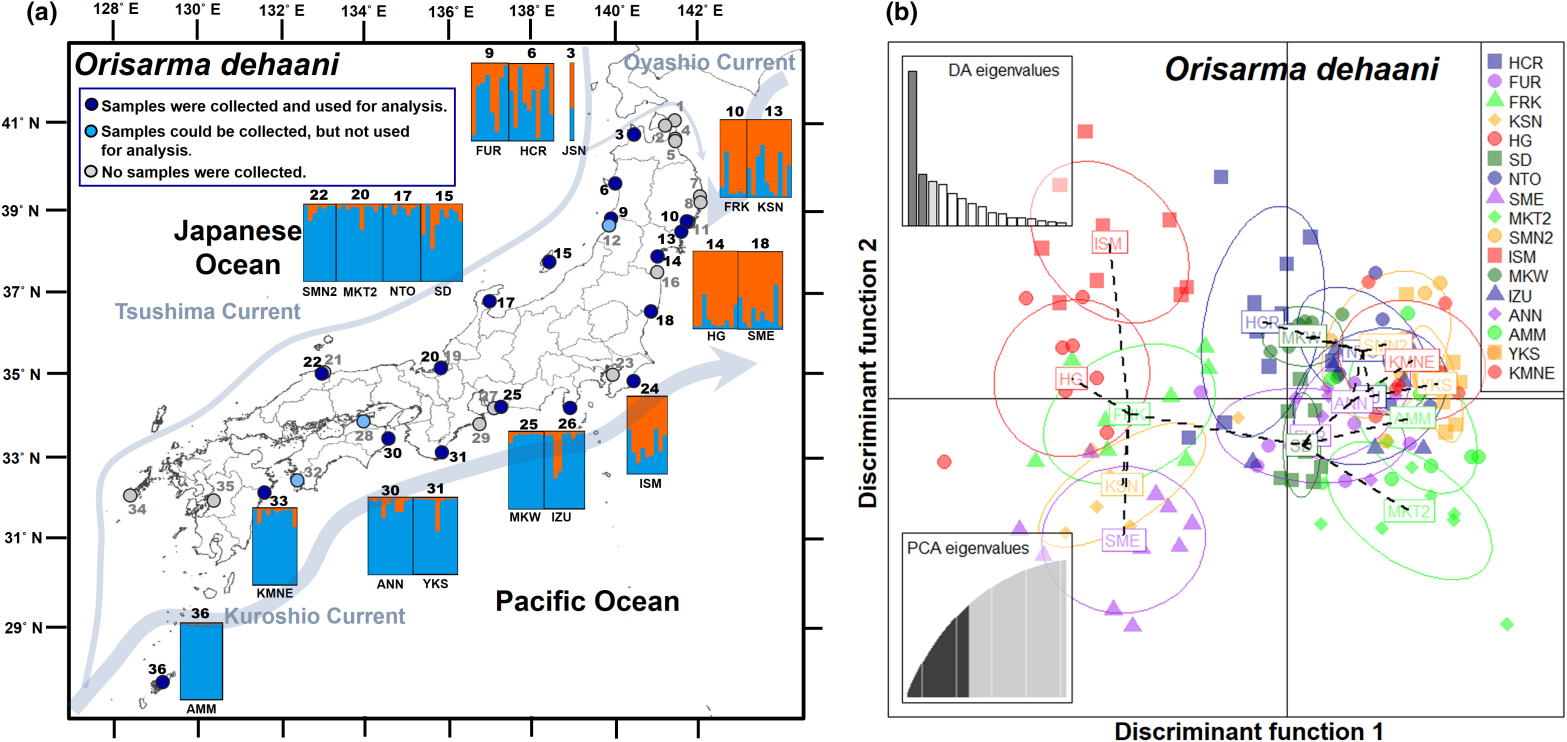
Results of Bayesian analysis of population structure (STRUCTURE) with *K* = 2 clusters and discriminant analysis of principal components (DAPC) for 18 populations of *O*. *dehaani*. (a) STRUCTURE composition plot for each individual grouped by the sampling localities. The locality number and code from Table 1 are shown on the panels. (b) Plots of the first two axes obtained by DA. Individuals from each population are represented by a population‐specific symbol. The number of PCs retained, as determined by the a‐score, was 58. Eigenvalues of the PCA and DA are inserted.

After removing extremely short reads and low‐quality reads, 17,536,469 reads (113,873 ± 2653 reads per sample) were obtained from 19,215,562 raw reads (124,776 ± 2870 reads per sample). A total of 1249 SNPs across 552 loci were obtained from 154 individuals taken from 18 populations of *Orisarma dehaani*. *H*o ranged from 0.158 to 0.191, while *H*e ranged from 0.009 to 0.258. *F*_IS_ was positive in all but one population (range: 0.261 to 0.392; mean = 0.326) (Table 1). The STRUCTURE analysis of the MIG‐seq data, which supported the partitioning of *O*. *dehaani* into two genomic components (maximum Δ*K* = 201.570: at *K* = 2; Figure S3a), showed a clear distinction between 5 local populations on the Pacific coast of the Tohoku region and 13 local populations on other Japanese coasts (Figure 1a). The same result was obtained by the DAPC analysis, which showed that the first axis separated the Tohoku Pacific coast populations from the other populations (Figure 1b). In this analysis, the populations of these two regions separated from each other with 96% probability, indicating that *O*. *dehaani* were grouped into two phylogenetic groups (Figure S4). The relative directional migration rates from the Pacific coast of the Tohoku region (cluster 1) to other Japanese coasts (cluster 2), detected by STRUCTURE analysis, were 0.5, while the rate in the opposite direction was 1.0 (Figure S5). Although the bootstrap analysis showed that the rate of the former diction was not significant (*p* > .05), the rate of the latter direction was significant (*p* < .001).

**TABLE 1:** Genetic diversity parameters of the 18 *O. dehaani* populations in the Japanese archipelago estimated from 1249 single nucleotide polymorphisms (SNPs).

Locality number	Locality		Prefecture	*N*	*H*o	*H*e	*F*_IS_	Latitude	Longitude
3	JSN	Jusanko	Aomori	1	0.18	0.009	NA	41.0317	140.4085
6	HCR	Hachiro‐Egawa R.	Akita	10	0.191	0.249	0.316*	39.8883	139.9702
9	FUR	Fukura R.	Yamagata	8	0.172	0.235	0.392*	39.0708	139.8735
10	FRK	Furukawa‐numa (lagoon)	Iwate	10	0.183	0.253	0.354*	39.0087	141.6395
13	KSN	Tsuya R., Kesen‐numa City	Miyagi	6	0.178	0.213	0.334*	38.7646	141.5125
14	HG	Hagashi‐yachi Lagoon, Sendai City	Miyagi	10	0.186	0.25	0.346*	38.1856	140.9595
15	SD	Sado Island	Niigata	9	0.173	0.216	0.305*	38.0626	138.4306
17	NTO	Notojima	Ishikawa	8	0.17	0.205	0.307*	37.1447	137.0412
18	SME	Samegawa R.	Fukushima	10	0.189	0.258	0.345*	36.9037	140.8056
20	MKT2	Mikata, Lake‐Suga	Fukui	10	0.189	0.237	0.28*	35.5802	135.9002
22	SMN2	Ohashi R.	Shimane	7	0.166	0.208	0.333*	35.4519	133.1230
24	ISM	Isumi R.	Chiba	9	0.183	0.247	0.355*	35.2823	140.4014
25	MKW	Mikawa Bay, Shiokawa R.	Aichi	8	0.158	0.191	0.302*	34.6785	137.3068
26	IZU	Ogamo R., Izu Pen.	Shizuoka	9	0.159	0.205	0.338*	34.6555	138.9182
30	ANN	Nakagawa R., Anan City	Tokushima	10	0.18	0.235	0.313*	33.9387	134.6760
31	YKS	Yukashi Lagoon	Wakayama	10	0.158	0.214	0.343*	33.6173	135.9245
33	KMNE	Kumanoe R.	Miyazaki	10	0.178	0.234	0.32*	32.6784	131.7826
36	AMM	Amami Island, Sumiyo R.	Kagoshima	9	0.165	0.199	0.261*	28.2581	129.4097
Mean					0.175	0.214	0.326		

*Note*: Significant deviation from Hardy–Weinberg equilibrium at *p* < .05 is denoted by asterisk.

### 3.2 Chiromantes haematocheir

#### 3.2.1 Mitochondrial DNA

In this species, DNA segments of 499 bp in the mitochondrial COI gene were commonly sequenced with no insertions and no deletions. In these segments, we found a total of 39 variable sites and identified 50 haplotypes. Twelve of the 50 haplotypes were shared among 2 to 19 localities, while the remaining 38 haplotypes were locality‐specific (Table S3). Haplotype diversity values (*h*) and nucleotide diversity values (*π*) ranged from 0.533 to 0.917 and from 0.0015 to 0.0067, respectively. According to a statistically parsimonious haplotype network (Figure S6a), the dominant haplotype was Ch1, and the other haplotypes were separated from Ch1 by 1 to 6 mutational steps, and formed three “star‐shaped” clusters (Figure S6a,b).

#### 3.2.2 MIG‐seq

**FIGURE 2:**
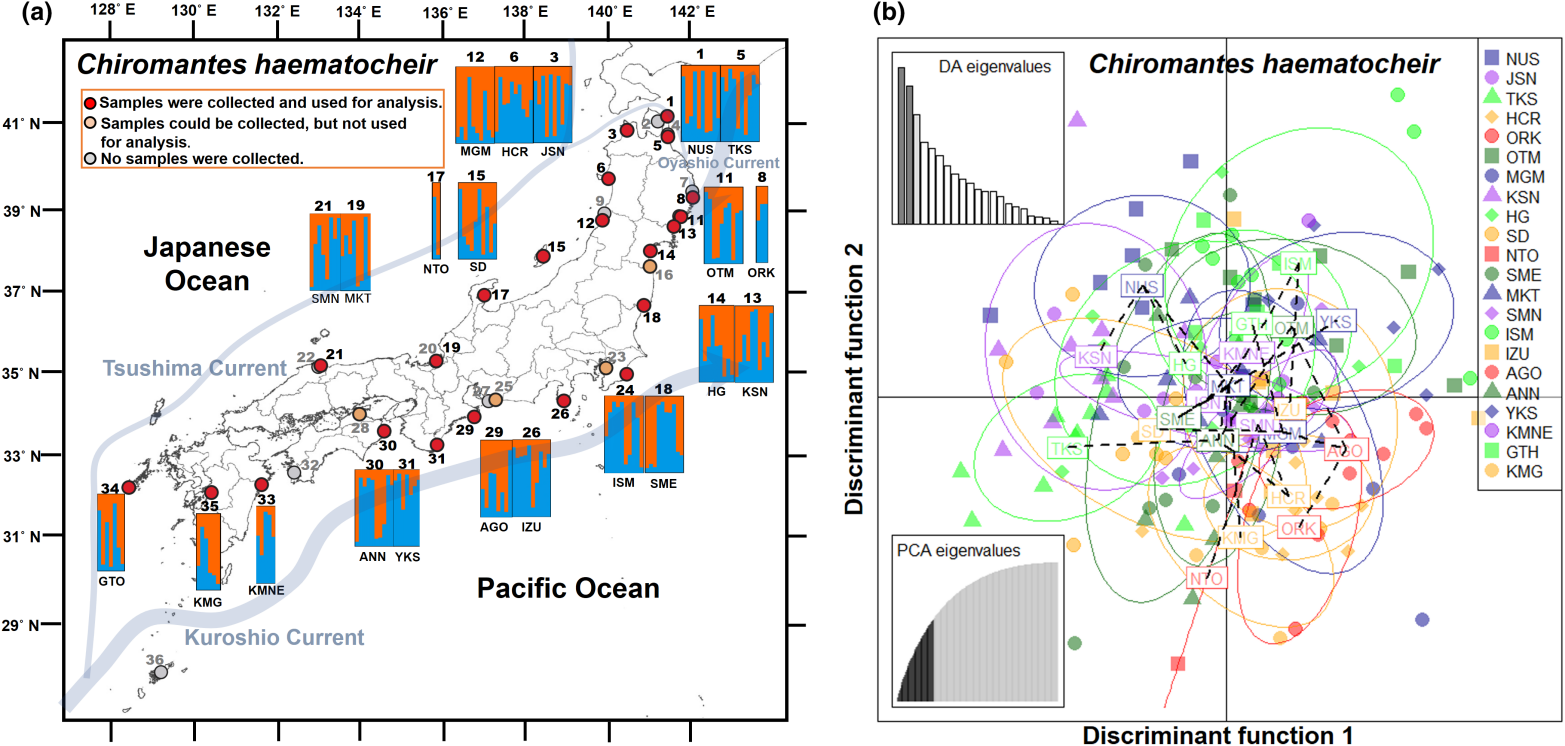
Results of Bayesian analysis of population structure (STRUCTURE) with *K* = 2 clusters and discriminant analysis of principal components (DAPC) for 21 populations of *C*. *haematocheir*. (a) STRUCTURE composition plot for each individual grouped by the sampling localities. The locality number and code from Table 2 are shown on the panels. (b) Plots of the first two axes obtained by DA. Individuals from each population are represented by a population‐specific symbol. The number of PCs retained, as determined by the a‐score, was 41. Eigenvalues of the PCA and DA are inserted.

After removing extremely short reads and low‐quality reads, 17,859,099 reads (98,591 ± 2264 reads per sample) were obtained from 19,585,132 raw reads (107,023 ± 2446 reads per sample). We found 583 SNPs across 285 loci in 183 individuals taken from 22 *C. haematocheir* populations. In this species, *H*o and *H*e ranged from 0.18 to 0.234 and 0.146 to 0.281, respectively. *F*_IS_ was positive in all populations (range: 0.193 to 0.306; mean = 0.238) (Table 2). The STRUCTURE analysis of the MIG‐seq data did not clearly divide the populations into genetically distinct groups (maximum Δ*K* = 6.287: at *K* = 2; Figure 2a; Figure S3b). The same result was obtained by the DAPC analysis, which showed overlap among the populations in the coasts of the Japanese archipelago (Figure 2b).

**TABLE 2:** Genetic diversity parameters of the 21 *C. haematocheir* populations in the Japanese archipelago estimated from 583 single nucleotide polymorphisms (SNPs).

Locality number	Locality		Prefecture	*N*	*H*o	*H*e	*F*_IS_	Latitude	Longitude
1	NUS	Noushi R.	Aomori	10	0.217	0.257	0.248*	41.3611	141.3591
3	JSN	Jusanko	Aomori	10	0.23	0.274	0.241*	41.0317	140.4085
5	TKS	Takase R.	Aomori	10	0.219	0.265	0.256*	40.8789	141.3730
6	HCR	Hachiro‐Egawa R.	Akita	10	0.227	0.259	0.193*	39.8883	139.9702
8	ORK	Orikasa R.	Iwate	3	0.18	0.169	0.306*	39.4478	141.9608
11	OTM	Otomoura Inlet	Iwate	10	0.228	0.263	0.216*	38.9947	141.6816
12	MGM	Mogami R.	Yamagata	10	0.207	0.239	0.221*	38.9109	139.8272
13	KSN	Tsuya R., Kesen‐numa City	Miyagi	10	0.228	0.261	0.207*	38.7646	141.5125
14	HG	Hagashi‐yachi Lagoon, Sendai City	Miyagi	9	0.23	0.278	0.254*	38.1856	140.9595
15	SD	Sado Island	Niigata	10	0.23	0.274	0.231*	38.0626	138.4306
17	NTO	Notojima	Ishikawa	2	0.226	0.146	0.158	37.1447	137.0412
18	SME	Samegawa R.	Fukushima	10	0.21	0.264	0.289*	36.9037	140.8056
19	MKT	Mikata, Lake‐Kugushi	Fukui	8	0.222	0.246	0.237*	35.5802	135.9002
21	SMN	Ooejima, Lake‐Shinzi	Shimane	8	0.216	0.255	0.29*	35.4855	133.1796
24	ISM	Isumi R.	Chiba	10	0.234	0.272	0.209*	35.2823	140.4014
26	IZU	Ogamo R., Izu Pen.	Shizuoka	10	0.229	0.281	0.249*	34.6555	138.9182
29	AGO	Zaga Island, Ago Bay	Mie	8	0.228	0.245	0.195*	34.2758	136.8047
30	ANN	Nakagawa R., Anan City	Tokushima	10	0.21	0.257	0.261*	33.9387	134.6760
31	YKS	Yukashi Lagoon	Wakayama	7	0.218	0.236	0.219*	33.6173	135.9245
33	KMNE	Kumanoe R.	Miyazaki	5	0.204	0.178	0.238*	32.6784	131.7826
34	GTO	Fukue Island, Goto Islands	Nagasaki	7	0.212	0.252	0.292*	32.6089	128.6510
35	KMG	Kumagawa R.	Kumamoto	6	0.221	0.221	0.218*	32.4925	130.5996
Mean					0.219	0.245	0.238		

*Note*: Significant deviation from Hardy–Weinberg equilibrium at *p* < .05 is denoted by asterisk.

### 3.3 Helice tridens

#### 3.3.1 Mitochondrial DNA

In this species, DNA segments of 490 bp in the mitochondrial COI gene were commonly sequenced with no insertions and no deletions. In these segments, we found a total of 30 variable sites different and 35 mtDNA haplotypes of *H. tridens*. Among them, 14 haplotypes were found more than 2 localities, while the other 21 haplotypes were locality‐specific (Table S4). The haplotype diversity values (*h*) and nucleotide diversity values (*π*) ranged from 0.533 to 0.917 and from 0.0012 to 0.0047, respectively. According to a statistically parsimonious haplotype network (Figure S7a), the dominant haplotype was Ht1, and the other haplotypes were separated from Ht1 by 1 to 2 mutational steps, and formed three “star‐shaped” clusters (Figure S7a,b).

#### 3.3.2 MIG‐seq

**FIGURE 3:**
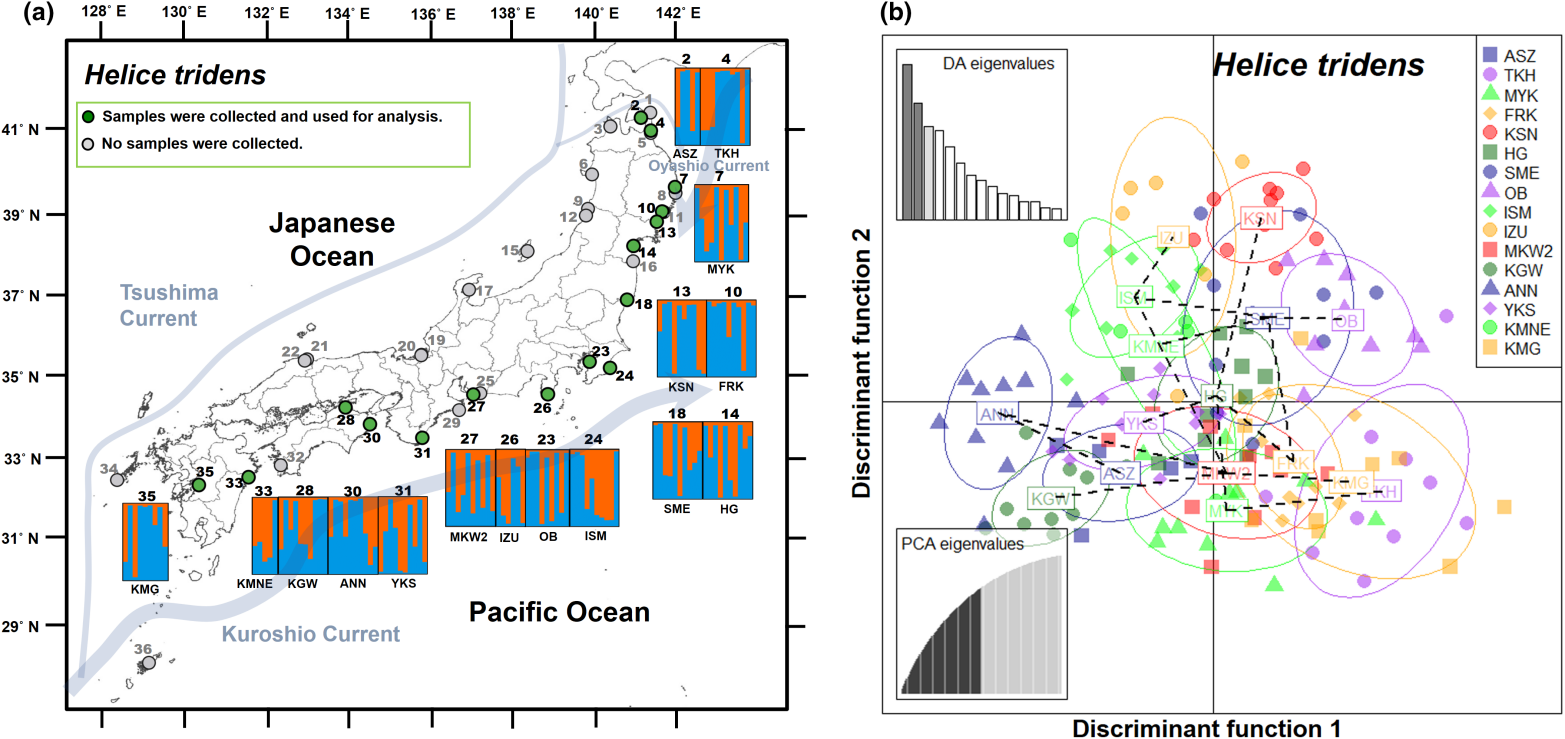
Results of Bayesian analysis of population structure (STRUCTURE) with *K* = 2 clusters and discriminant analysis of principal components (DAPC) for 16 populations of *H*. *tridens*. (a) STRUCTURE composition plot for each individual grouped by the sampling localities. The locality number and code from Table 3 are shown on the panels. (b) Plots of the first two axes obtained by DA. Individuals from each population are represented by a population‐specific symbol. The number of PCs retained, as determined by the a‐score, was 72. Eigenvalues of the PCA and DA are inserted.

After removing extremely short reads and low‐quality reads, 15,748,543 reads (108,611 ± 2055 reads per sample) were obtained from 17,180,320 raw reads (118,485 ± 2212 reads per sample). We found 1025 SNPs across 539 loci from 145 individuals taken from 16 *H. tridens* populations. *H*o and *H*e ranged from 0.207 to 0.235 and 0.216 to 0.268, respectively. *F*_IS_ was positive in all populations (range: 0.117 to 0.26; mean = 0.188) (Table 3). In the STRUCTURE analysis, the population did not genetically separate the population*s* (maximum Δ*K* = 38.365: at *K* = 2; Figure 3a; Figure S3c). This trend was confirmed by the DAPC analysis, which showed overlap between the populations (Figure 3b).

**TABLE 3:** Genetic diversity parameters of the 16 *H. tridens* populations in the Japanese archipelago estimated from 1025 single nucleotide polymorphisms (SNPs).

Locality number	Locality		Prefecture	*N*	*H*o	*H*e	*F*_IS_	Latitude	Longitude
2	ASZ	Ashizaki	Aomori	5	0.231	0.218	0.117*	41.2400	141.1341
4	TKH	Takahoko‐numa (lagoon)	Aomori	10	0.232	0.256	0.161*	40.9318	141.3726
7	MYK	Tsugaruishi R., Miyako Bay	Iwate	11	0.214	0.256	0.233*	39.5872	141.9471
10	FRK	Furukawa‐numa (lagoon)	Iwate	10	0.221	0.256	0.203*	39.0087	141.6395
13	KSN	Tsuya R., Kesen‐numa City	Miyagi	10	0.229	0.268	0.222*	38.7646	141.5125
14	HG	Hagashi‐yachi Lagoon, Sendai City	Miyagi	10	0.232	0.251	0.146*	38.1856	140.9595
18	SME	Samegawa R.	Fukushima	10	0.226	0.257	0.195*	36.9037	140.8056
23	OB	Obitsu R.	Chiba	9	0.216	0.249	0.205*	35.4290	139.9121
24	ISM	Isumi R.	Chiba	10	0.235	0.267	0.186*	35.2823	140.4014
26	IZU	Ogamo R., Izu Pen.	Shizuoka	6	0.221	0.243	0.26*	34.6555	138.9182
27	MKW2	Mikawa Bay, Atsumi Bay	Aichi	10	0.207	0.242	0.222*	34.6446	137.1417
28	KGW	Shinkawa R.	Kagawa	10	0.234	0.262	0.167*	34.3399	134.0941
30	ANN	Nakagawa R., Anan City	Tokushima	10	0.232	0.251	0.138*	33.9387	134.6760
31	YKS	Yukashi Lagoon	Wakayama	10	0.219	0.264	0.24*	33.6173	135.9245
33	KMNE	Kumanoe R.	Miyazaki	5	0.225	0.216	0.173*	32.6784	131.7826
35	KMG	Kumagawa R.	Kumamoto	9	0.231	0.242	0.133*	32.4925	130.5996
Mean					0.225	0.250	0.188		

*Note*: Significant deviation from Hardy–Weinberg equilibrium at *p* < .05 is denoted by asterisk.

### 3.4 A comparison of the spatial genetic structure of three semi‐terrestrial crabs

Pairwise *F*_ST_ comparisons of *O*. *dehaani* showed significant difference in the population genetics between the Pacific coast of the Tohoku region and other Japanese coasts (Table S5). For *C*. *haematocheir*, pairwise *F*_ST_ values were significant between the southern Kyushu Island population (locality code KMNE) and the other populations (Table S6). The KMNE population showed significant *F*_ST_ values against 7 of the 21 populations examined. The pairwise *F*_ST_ values of *H. tridens* were not significant among the coastal populations of Japan, except for five pairs of populations (Table S7).

Pairwise genetic distance (*F*_ST_/(1 − *F*_ST_)) was significantly and positively correlated with the pairwise geographic distance in *O*. *dehaani* (*p* = .0056, *r* = .296), whereas no such significant correlation was found in *C*. *haematocheir* (*p* = .1634 and *r* = .080) *and H*. *tridens* (*p* = .2496 and *r* = .089) (Figure S8, Tables S8–S10).

We also found that the inbreeding coefficient (*F*_IS_) was significantly higher in *O*. *dehaani* (0.326) than in *C*. *haematocheir* (0.238) and *H*. *tridens* (0.188) (Figure S9). Furthermore, the *F*_IS_ of *O*. *dehaani* populations was significantly higher on the Pacific coast of the Tohoku region (*F*_IS_ = 0.347) compared to the other Japanese coasts (*F*_IS_ = 0.318) (Figure S10).

## 4 DISCUSSION

The present study showed that *O. dehaani* populations were genetically divided into two groups, i.e. those distributed along the Pacific coast of the Tohoku region (from FRK to ISM populations) and those distributed along other Japanese coasts. However, *C. haematocheir* and *H. tridens* populations did not show such a clear regional differentiation in genetic structure. Below, we first discuss why the spatial pattern of population genetic structure differed among these three species. We then discuss whether or not there are populations at risk of extinction.

### 4.1 Effects of life history traits

The present study showed that, among the three semi‐territorial crabs, *O*. *dehaani* had a significantly higher inbreeding coefficient than *C*. *haematocheir* and *H*. *tridens*. Many benthic marine organisms have pelagic larval stages that extend their range by drifting in ocean currents as plankters (Cowen & Sponaugle, 2009). In these organisms, the distance of the dispersal range is generally related to the duration of the planktonic larval stages (Levin, 2006; Shanks, 2009). In *C. haematocheir* and *H. tridens*, zoeal stages I to V are planktonic, whereas in *O. dehaani* only zoeal stages I to IV are planktonic (Cuesta et al., 2006; Mia & Shokita, 1997; Terada, 1976). The number of planktonic zoeal stages seems to correspond to the pelagic larval period. In fact, Terada (1976) reported that the pelagic larval period was 17 and 15 days for *C. haematocheir* and *H. tridens*, respectively, while it was 12 days for *O. dehaani*. This observation suggests that *O. dehaani* has the lowest dispersal ability among the three semi‐terrestrial crabs.

Saigusa (1981) showed that *C. haematocheir* and *H. tridens* released larvae in a clear semi‐annual rhythm, whereas the larval release activity of *O. dehaani* did not have such a clear semi‐annual rhythm. The observations suggest that *O. dehaani* larvae do not need to migrate offshore and, therefore, remain in nearshore waters (Baba, 1978; Saigusa, 1981). In support of this, *O. dehaani* larvae are more tolerant to low salinities than *C. haematocheir* and *H. tridens* larvae, supporting the idea that they complete their life cycle without migrating offshore (Irawan & Kijima, 1994; Matsumoto et al., 2020). If these reproductive and developmental traits limit the migratory range of the larvae, that may have driven the observation that *O*. *dehaani* populations are genetically differentiated into two biogeographically distinct groups.

However, the low dispersal ability of *O. dehaani* larvae cannot explain why the genetic structure was different between populations on the Pacific coast of the Tohoku region and those on other Japanese coasts. If their population genetic structure is determined by low dispersal ability alone, it is likely that the genetic structure is fragmented at different locations along the coast of the Japanese archipelago. Thus, their population genetic structure should be additionally shaped by other factors, such as ocean currents, which may limit larval transport and dispersal (Williams & Hastings, 2013).

### 4.2 Effects of ocean current and temperature

It is well known that the Pacific Ocean from the Tohoku region to the east coast of the Boso Peninsula has a lower surface water temperature than other areas of Japan, except for the coasts of Hokkaido, due to the influence of the Oyashio Current (Nishimura, 1992). *Orisarma dehaani* was not distributed along the coast of Hokkaido, which is strongly influenced by the Oyashio Current (Miyake, 1983). Lake Jusanko on the Sea of Japan side in northwestern Tohoku is the northern distribution limit of this species (Yuhara & Suzuki, 2021; this study). On the Pacific coast side, we could not collect *O. dehaani* individuals at the sampling localities located at latitudes higher than Furukawa‐Numa Lagoon, i.e., NUS, ASZ, TKH, TKS, MYK, ORK, and OTM (Yuhara & Suzuki, 2021; this study). The result suggests that the northern limit of their range was limited by low temperature. Since the Pacific coasts of the Tohoku region are still under the influence of the Oyashio Current, *O. dehaani* populations in the Tohoku region may have adapted to relatively low temperature, as in the case of the goby fish *Chaenogobius annularis* and the abalone *Haliotis discus*, which have different genetic structures in the northeastern areas of the Japanese Pacific coast due to the influence of low seawater temperature originating from the Oyashio Current (Hirase et al., 2021; Hirase & Ikeda, 2015). The Kuroshio Current acts as a conveyor for larvae of various tropical and subtropical brachyuran crabs to the high latitude regions of Japan (Nishimura, 1992). However, it leaves the coast at the Boso Peninsula and flows eastward as the Kuroshio Extension in central Japan at about 35° N (Qiu, 2001). Thus, this geographic pattern of the Kuroshio Current appears to functionally act as a barrier to genetically separate *O. dehaani* populations on the Pacific coast of Tohoku from those in other regions.

### 4.3 Local isolation

For *C. haematocheir* and *H. tridens*, neither mtDNA nor MIG‐Seq analysis detected genetic separation between populations living along the coasts of the Japanese archipelago. However, *C. haematocheir* was genetically distinct from other local populations in the Kumanoe River population (KMNE) in Miyazaki Prefecture. One possible reason for this local genetic differentiation is the effect of population size at the edge of the range. The southern limit of the distribution of *C. haematocheir* in the Japanese archipelago is in Kagoshima Prefecture (Osawa, 2022; Schubart & Ng, 2020), and the Kumanoe River population (KMNE) is near the edge of its range. In general, peripheral populations exhibit low genetic diversity and greater genetic differentiation due to smaller effective population size and greater geographic isolation compared to geographically central populations (Eckert et al., 2008; Pandey & Rajora, 2012). In addition, genetic drift distinguishes the genetic structure of small populations from that of larger populations (Austerlitz et al., 1997; Signorile et al., 2014). Indeed, in Miyazaki Prefecture, where the KMNE population is located, *C. haematocheir* is listed as a “near‐threatened species” due to its small population size (Miyazaki Prefecture, 2020). The present study confirmed such local caution, as *C. haematocheir* populations in this marginal area of their range are somewhat genetically isolated and likely vulnerable to environmental changes. Note that these semi‐terrestrial crabs are also distributed in coastal areas of continental East Asia, which are located to the west and south of the Japanese archipelago. Therefore, it is possible that some of the local populations in the Japanese archipelago have the same genetic structure as the East Asian populations. However, the population genetic information of semi‐terrestrial crabs on the continental coasts is still limited. In future studies, it is necessary to clarify how the semi‐terrestrial crab populations in the Japanese Archipelago are genetically related to those in East Asia.

## 5 CONCLUSION

The present study revealed that *O. dehaani* shows little gene flow and clear genetic differentiation between populations in the Tohoku Pacific region and those on other Japanese coasts due to the influence of the Kuroshio and Oyashio currents. Thus, these Tohoku Pacific populations appear to be isolated and worthy of conservation as independently evolving population groups (i.e., Evolutionarily Significant Units, ESUs) (Moritz, 1999). In contrast, the genetic structures of *C. haematocheir* and *H. tridens* were not affected by ocean currents and were spatially homogeneous along the entire Japanese coast. However, for *C. haematocheir*, genetic differentiation was observed in a local population at the southern limit of its distribution range. Since its population is vulnerable due to its genetically small population size, it is necessary to continue monitoring changes in its population size in order to conserve a local population.

## AUTHOR CONTRIBUTIONS

**Takeshi Yuhara:** Conceptualization (lead); formal analysis (equal); funding acquisition (lead); investigation (equal); methodology (equal); writing – original draft (lead). **Hajime Ohtsuki:** Data curation (equal); formal analysis (equal); methodology (equal); writing – review and editing (equal). **Shun K. Hirota:** Data curation (equal); formal analysis (equal); methodology (equal); resources (equal); writing – review and editing (equal). **Yoshihisa Suyama:** Formal analysis (equal); methodology (equal); resources (equal); writing – original draft (equal). **Jotaro Urabe:** Conceptualization (lead); formal analysis (equal); funding acquisition (lead); methodology (equal); resources (equal); writing – original draft (equal); writing – review and editing (equal).

## CONFLICT OF INTEREST STATEMENT

The authors declare no conflict of interest.

## Supporting information


Data S1.

## Data Availability

All sequences newly obtained in this study were deposited in DDBJ under the accession numbers LC768188 to LC768321, and DRA016447.
