# Supplementary material for: Contrasting population genetic structure of three semi‐terrestrial brachyuran crabs on the coast of the Japanese archipelago
Source: Ecol Evol. 2024 Jun 5;14(6):e11484. doi: 10.1002/ece3.11484 (PMC11154805; doi:10.1002/ece3.11484)
Supplement: Supplementary file 1 — Data S1. [file ECE3-14-e11484-s001.docx]

Supporting information for the article

**Contrasting population genetic structure of three semi-terrestrial brachyuran crabs**

**on the coast of the Japanese archipelago**

Takeshi Yuhara, Hajime Ohtsuki, Shun K. Hirota, Yoshihisa Suyama, Jotaro Urabe


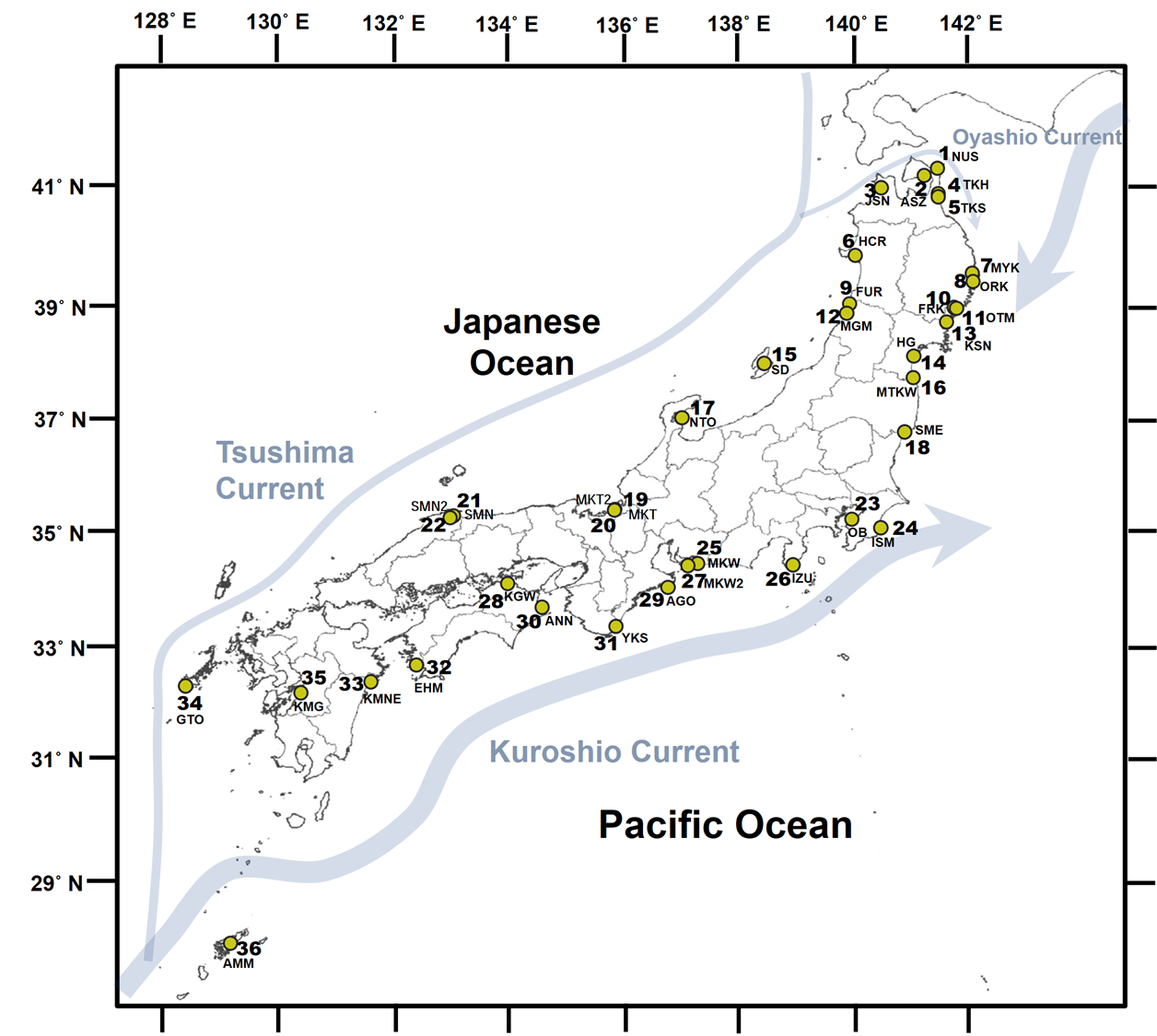


**Fig. S1.** Sampling localities and numbers of individuals of three semi-terrestrial crabs examined in the present study. 1: NUS, 2: ASZ, 3: JSN, 4: TKH, 5: TKS, 6: HCR, 7: MYK, 8: ORK, 9: FUR, 10: FRK, 11: OTM, 12: MGM, 13: KSN, 14: HG, 15: SD, 16: MTKW, 17: NTO, 18: SME, 19: MKT, 20: MKT2, 21: SMN, 22: SMN2, 23: OB, 24: ISM, 25: MKW, 26: IZU, 27: MKW2, 28: KGW, 29: AGO, 30: ANN, 31: YKS, 32: EHM, 33: KMNE, 34: GTO, 35: KMG. 36: AMM. See Tables S1 for more details.


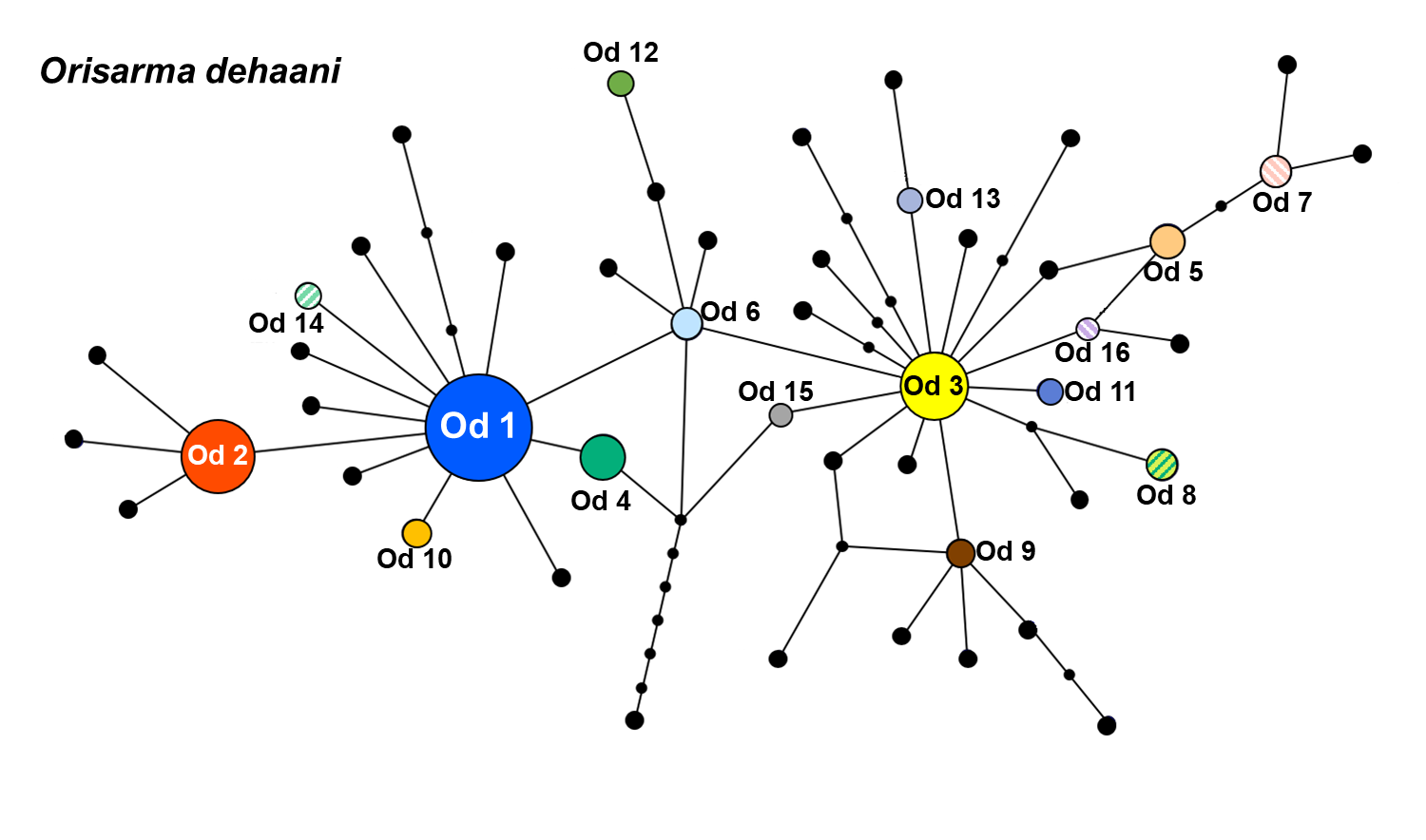

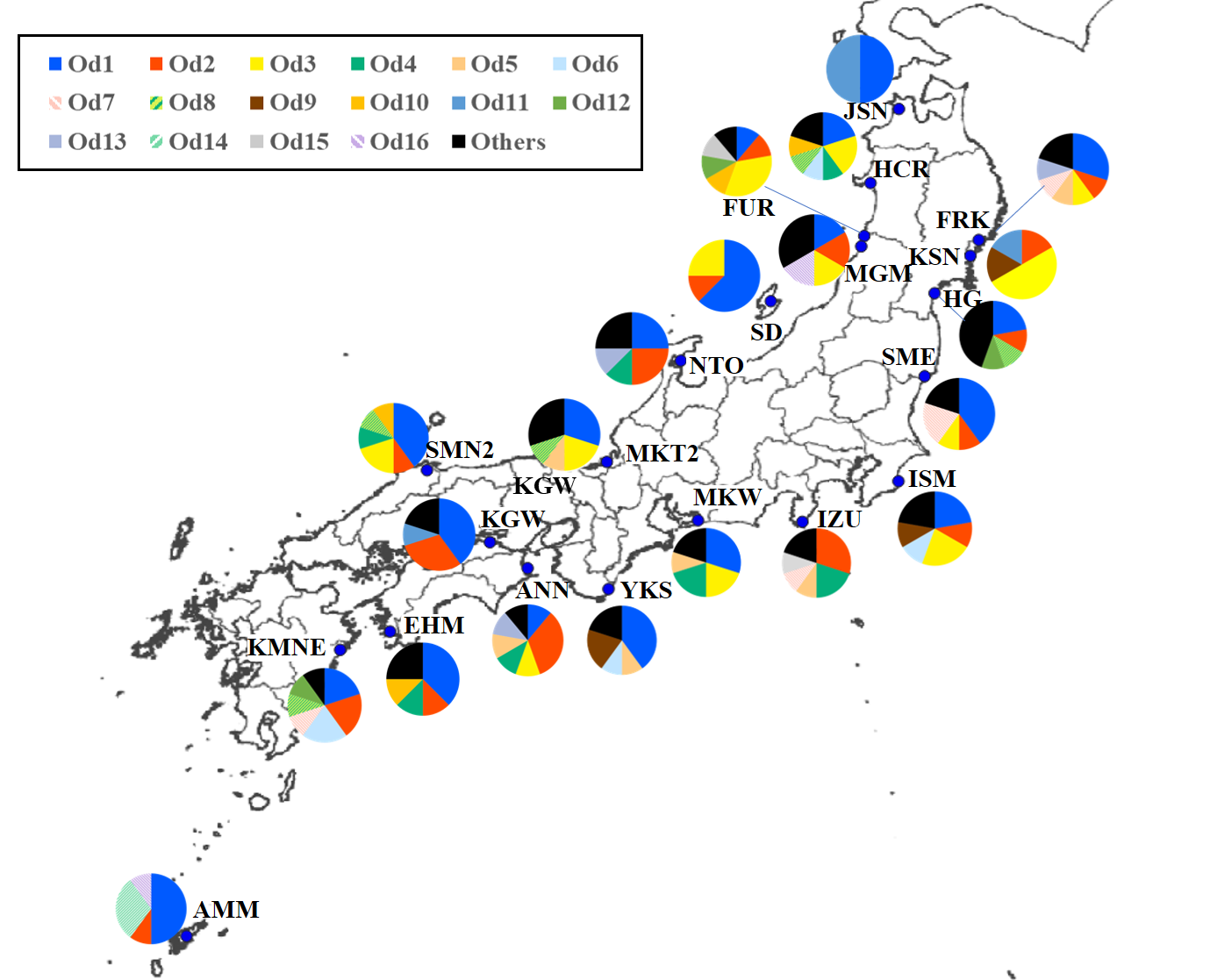


**b)**

**a)**

**Fig. S2.** Results of mtDNA COI haplotypes in *O. dehaani*. (a) Statistical parsimony network showing genetic relationships among haplotypes. Each circle represents one haplotype and its size is proportional to the number of individuals examined. The dominant haplotypes (Od1-Od14) correspond to those in Table S2. (b) Pie chart showing the frequency of different haplotypes at each sampling location. The different haplotypes are indicated by different colors.

**
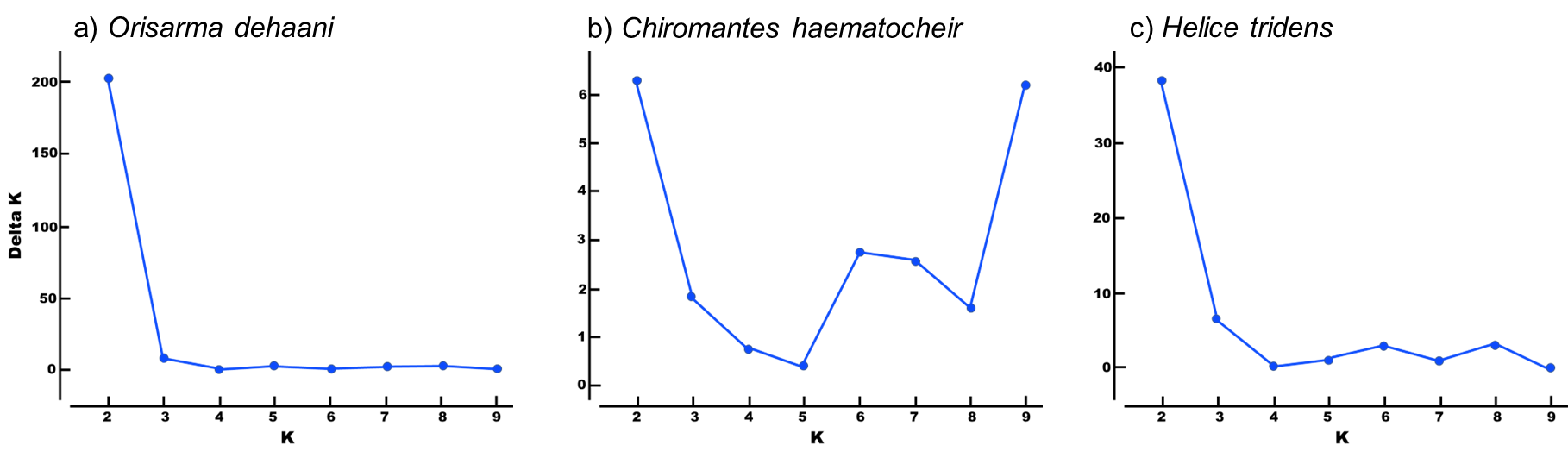
Fig. S3.** Delta K values plotted against the different cluster values (K=2 to 9) in STRUCTURE analysis for (a) *O. dehaani*, (b) *C. haematocheir*, and (c) *H. tridens* populations.


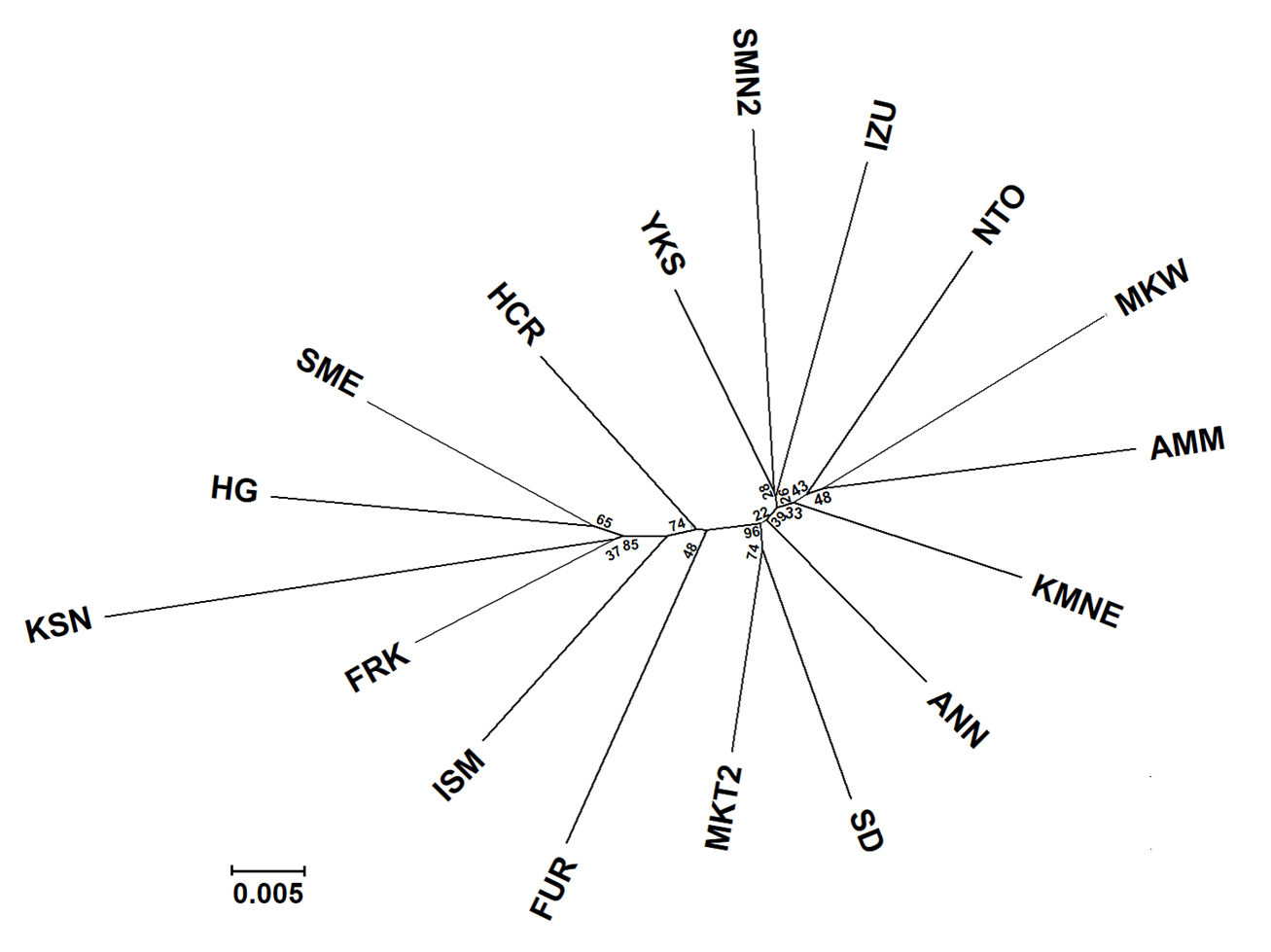


**Fig. S4.** Phylogenetic relationships among *O. dehaani* populations constructed from the DA distances using the neighbor-joining method. Bootstrap probabilities, estimated from 1000 replicates, are shown above the nodes.


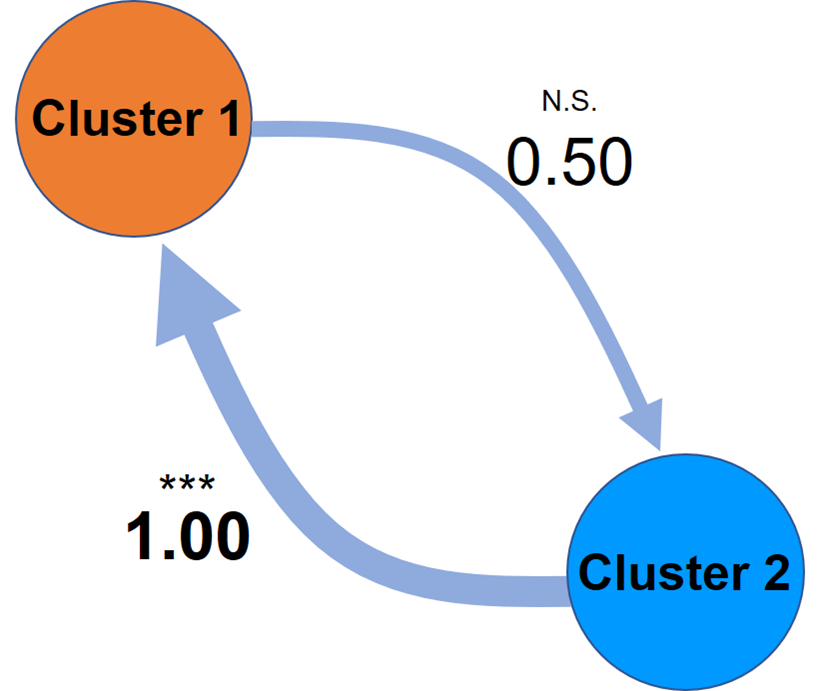


**Fig. S5.** Representation of asymmetric gene flow between *O. dehaani* populations on the Pacific coast of the Tohoku region (cluster 1) and other Japanese coasts (cluster 2) estimated by a method using div-MIGRATE online with *N*m and 1000 bootstrap permutations. The relative importance between the two directions is denoted by the width of the arrows.


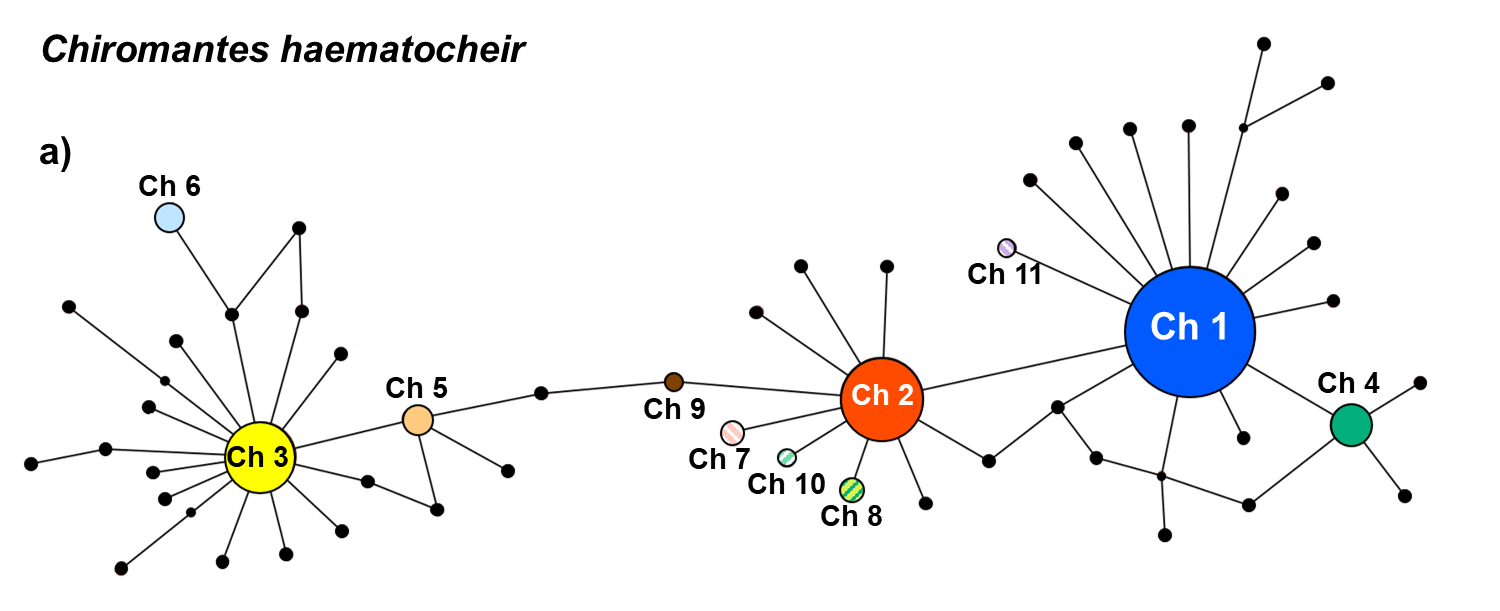


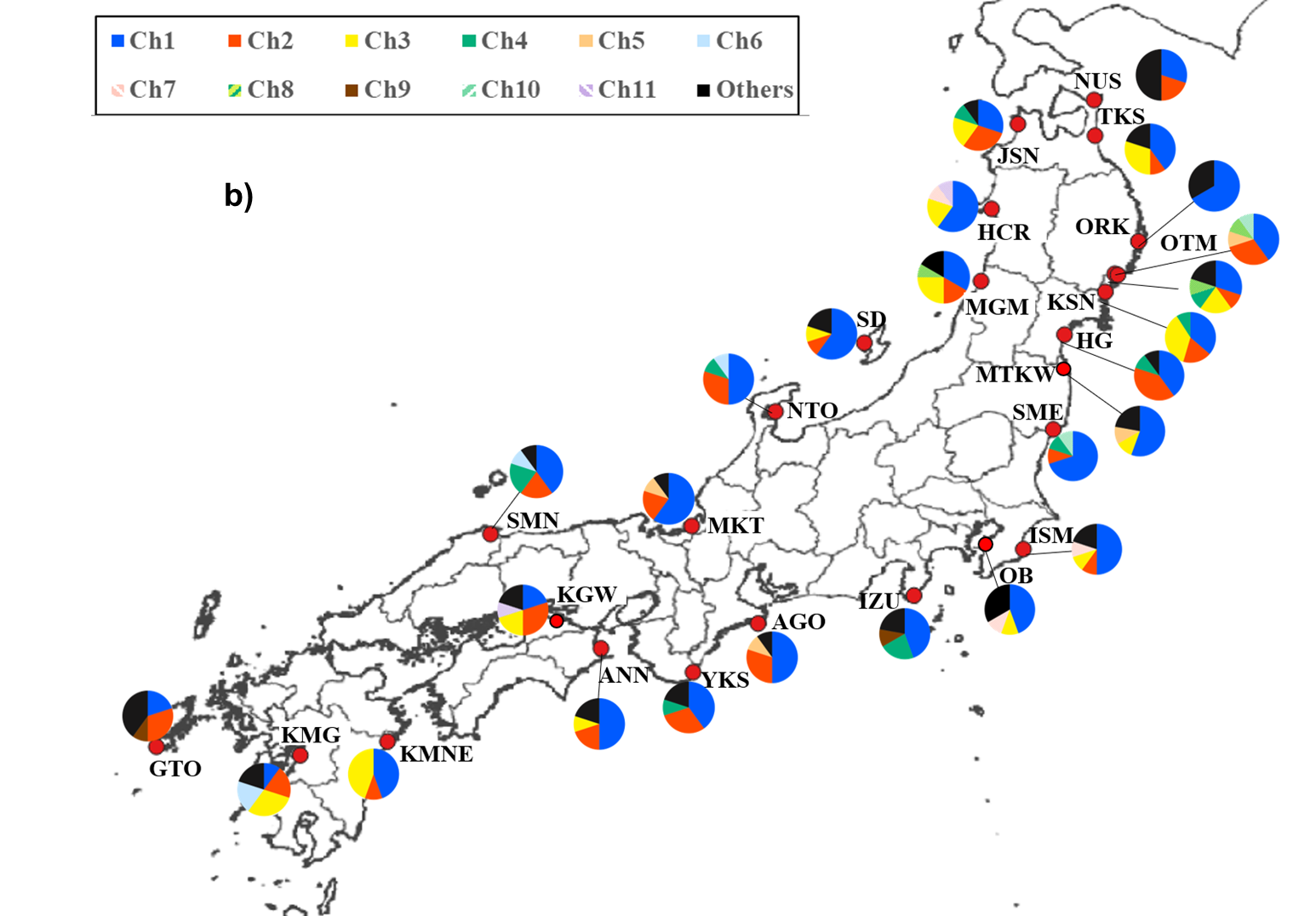


**Fig. S6** (a) Statistical parsimony network of mtDNA COI haplotypes from *C. haematocheir* specimens. Each circle represents a dominant haplotype. Dominant haplotypes (Ch1–Ch11) correspond to those in Table S3. The size of circles and squares is proportional to the number of haplotypes. (b) Sampling localities and pie charts represent mtDNA COI haplotype frequencies for *C*. *haematocheir*. See Tables S1 and S3 for more details.


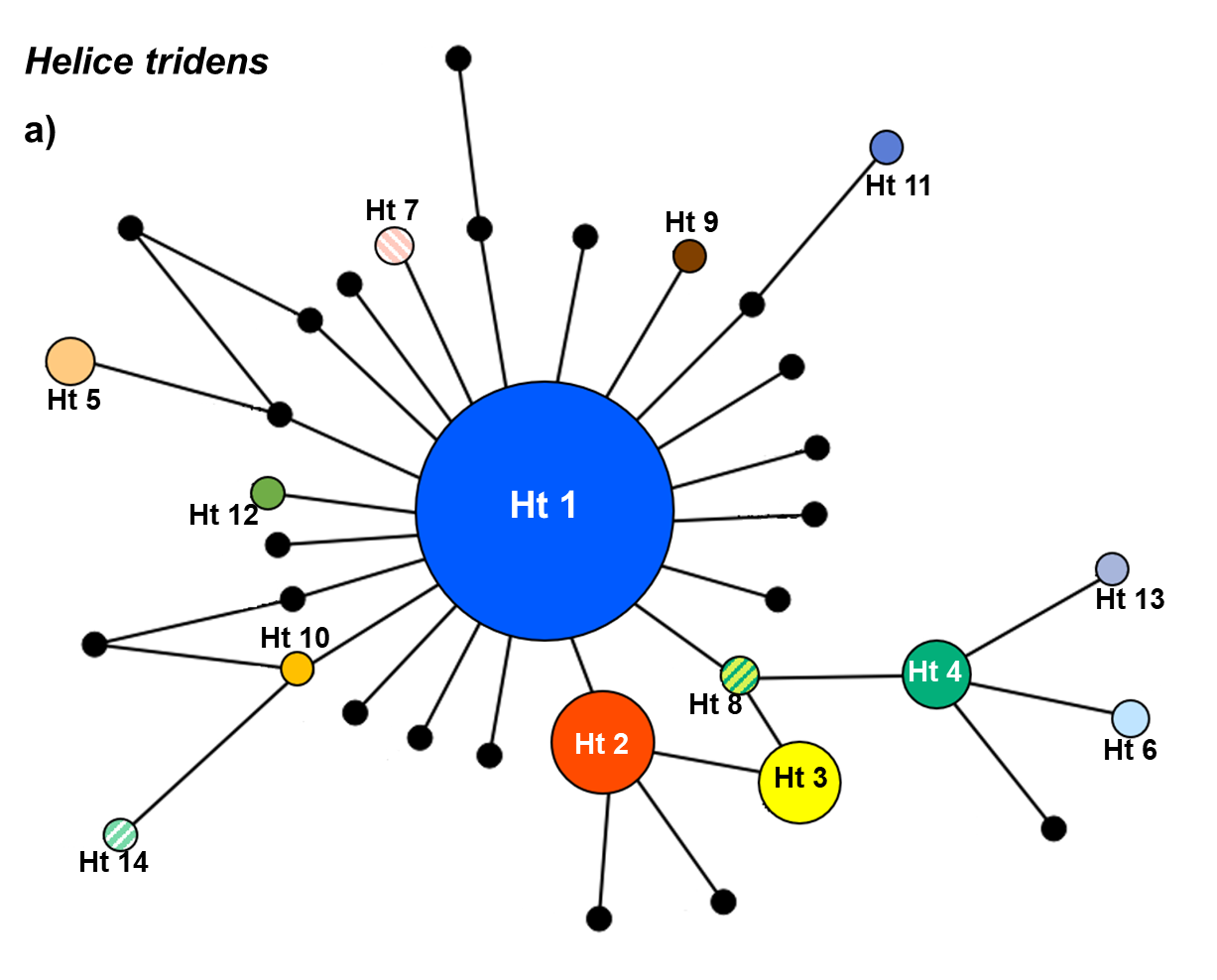


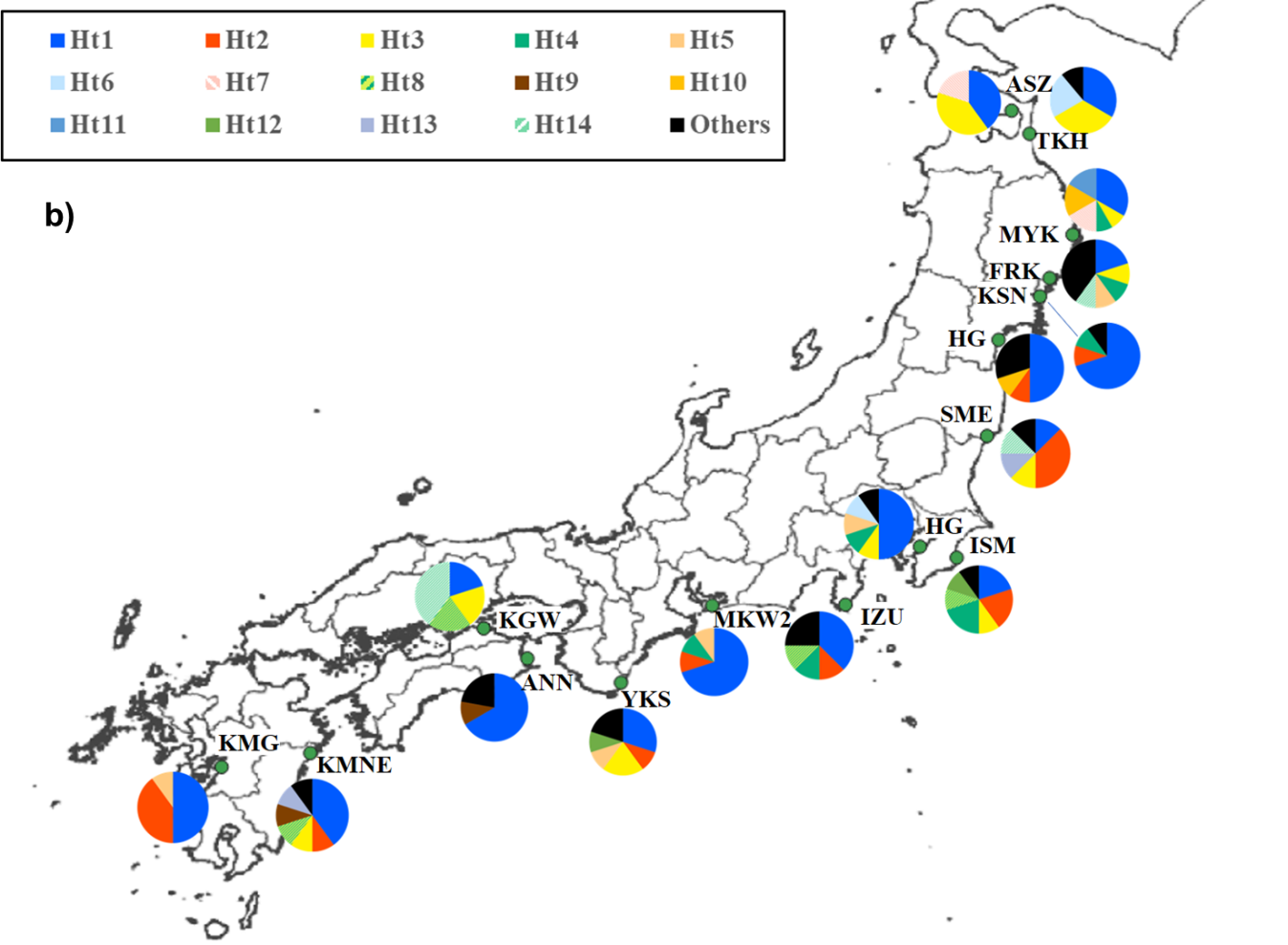


**Fig. S7** (a) Statistical parsimony network of mtDNA COI haplotypes from *H. tridens* specimens. Each circle represents a dominant haplotype. Dominant haplotypes (Ht1–Ht14) correspond to those in Table S4. The size of the circles and squares is proportional to the number of haplotypes. (b) Sampling localities and pie charts represent mtDNA COI haplotype frequencies for *H*. *tridens*. See Tables S1 and S4 for more details.


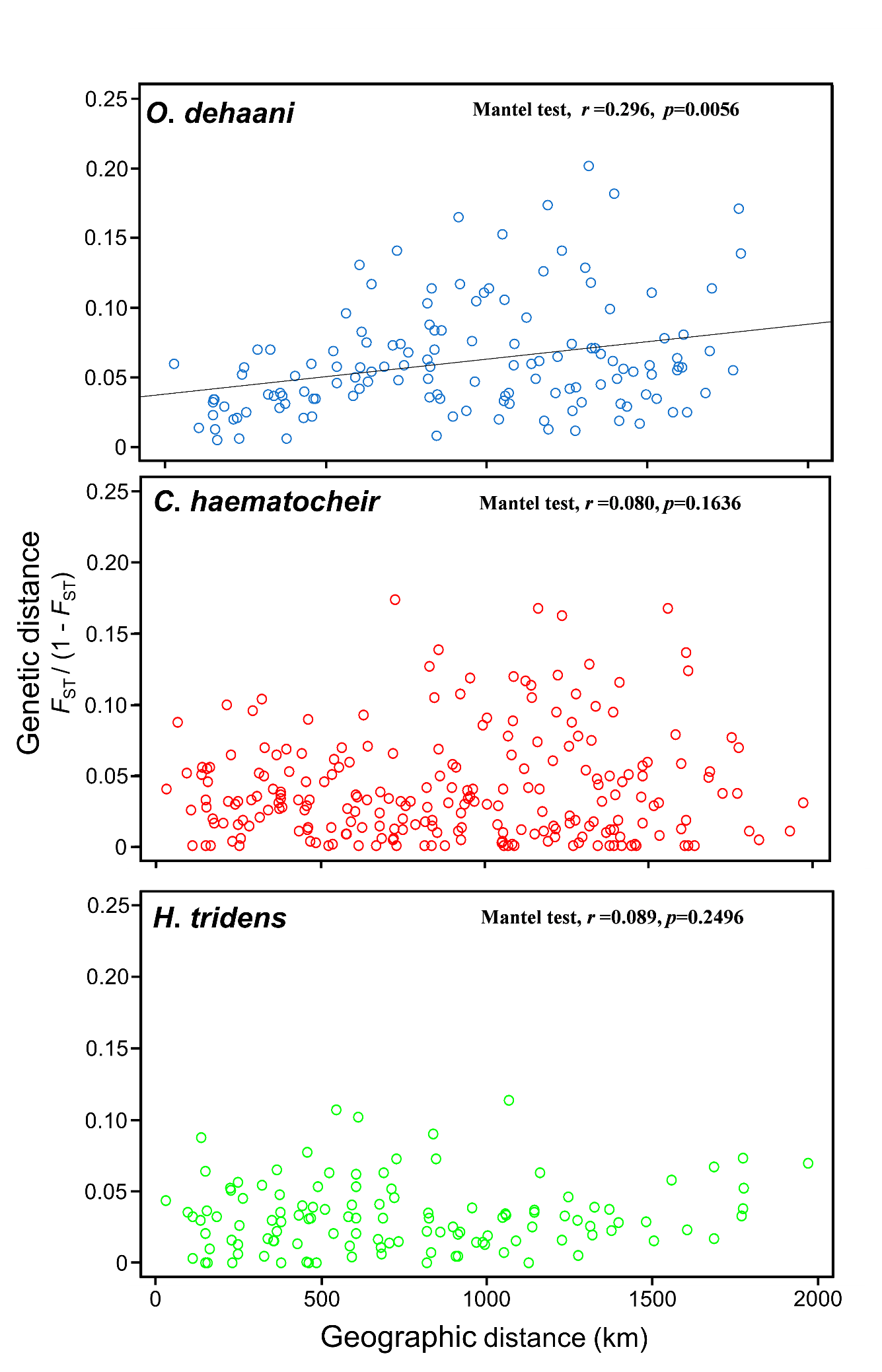


**Fig. S8.** Results of Mantel tests for three semi-terrestrial crabs (*O*. *dehaani*, *C*. *haematocheir*, and *H*. *tridens*) examining the effects of geographic distance on genetic distance (*F*_ST_/ (1 − *F*_ST_)).


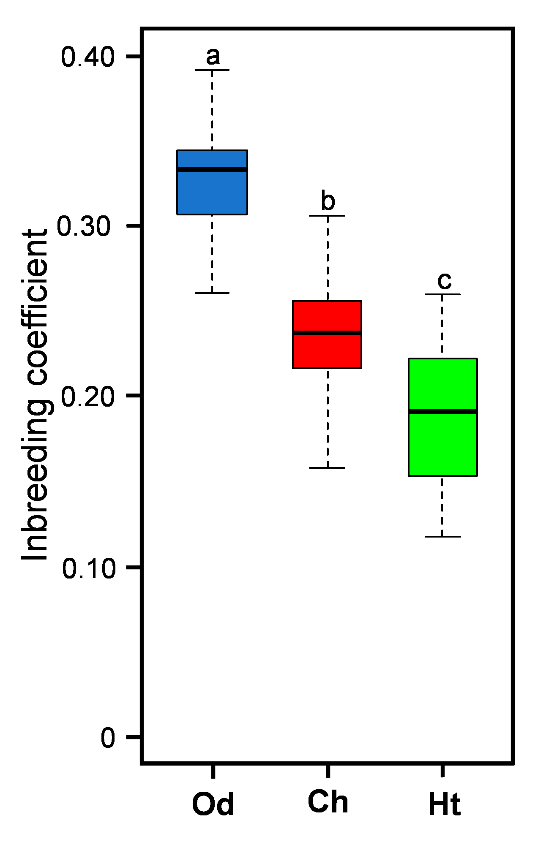


**Fig.S9.** Boxplots showing the inbreeding coefficient (*F*_IS_) values for each of the three semi-terrestrial crab populations (Od: *O. dehaani*; Ch: *C. haematocheir*; Ht: *H. tridens*). Significant differences in *F*_IS_ values between species are shown by different letters.


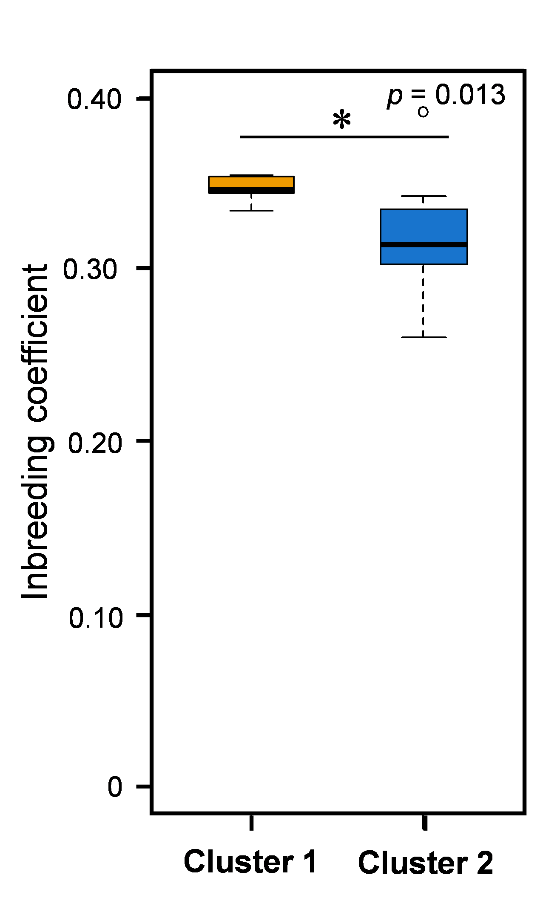


**Fig.S10.** Boxplots showing the inbreeding coefficient (*F*_IS_) for *O. dehaani* populations on the Pacific coast of the Tohoku region (cluster 1) and other Japanese coasts (cluster 2).

**Table S1.** Sampling localities and numbers of individuals of three semi-terrestrial crabs examined in the present study.


**Table S2.** Mitochondrial COI haplotype composition, haplotype diversity and nucleotide diversity of 18 *O*. *dehaani* populations.

**Table S3** Mitochondrial COI haplotype composition, haplotype diversity and nucleotide diversity of 21 *C*. *haematocheir* populations.

**Table S4.** Mitochondrial COI haplotype composition, haplotype diversity and nucleotide diversity of 16 *H*. *tridens* populations.

**Table S5.** Pairwise *F*_ST_ (lower diagonal) and *N*m (upper diagonal) values of *O*. *dehaani* populations. Probabilities of *F*_ST_ were calculated from 9999 replicates and corrected by Bonferroni correction for multiple tests (bold: significant at the *p* = 0.05 level).

**Table S6.** Pairwise *F*_ST_ (lower diagonal) and *N*m (upper diagonal) values of *C*. *haematochei*r populations. Probabilities of *F*_ST_ were calculated from 9999 replicates and corrected by Bonferroni correction for multiple tests (bold: significant at the *p* = 0.05 level).

**Table S7.**　Pairwise *F*_ST_ (lower diagonal) and *N*m (upper diagonal) values of *H*. *tridens* populations. Probabilities of *F*_ST_ were calculated from 9999 replicates and corrected by Bonferroni correction for multiple tests (bold: significant at the *p* = 0.05 level).

**Table S8.** Pairwise geographic distances of 18 locations for *O*. *dehaani* populations (km). Geographical distance was calculated as the nautical distance along the coasts between the pair of any two sites.

**Table S9.** Pairwise geographic distances of 21 locations for *C*. *haematocheir* populations (km). Geographical distance was calculated as the nautical distance along the coasts between the pair of any two sites.

**Table S10.** Pairwise geographic distances of 16 locations for *H*. *tridens* populations (km). Geographical distance was calculated as the nautical distance along the coasts between the pair of any two sites.
